# Performance of dual-layer spectral CT-based extracellular volume fraction to assess Ki-67 proliferation status in rectal cancer: comparison with diffusion kurtosis imaging

**DOI:** 10.1186/s13244-026-02376-4

**Published:** 2026-08-27

**Authors:** Zhuofeng Liang, Danfeng Wang, Liyan Tang, Fengping Chen, Anni Pan, Xiaohua Du, Xiaomin Liu, Zhijie Huang, Xian Liu, Weicui Chen

**Affiliations:** 1 Department of Radiology, The Second Affiliated Hospital of Guangzhou University of Chinese Medicine, Guangzhou, China; 2 Department of Radiology, Shantou Central Hospital, Shantou, China; 3 Department of Pathology, The Second Affiliated Hospital of Guangzhou University of Chinese Medicine, Guangzhou, China; 4 Clinical and Technical Support, Philips Healthcare, Guangzhou, China

**Keywords:** Rectal cancer, Ki-67, Diffusion kurtosis imaging, Extracellular volume fraction

## Abstract

**Objective:**

To evaluate and compare MRI-based diffusion kurtosis imaging (DKI), diffusion-weighted imaging (DWI), and dual-layer spectral CT (DLSCT)-derived extracellular volume (ECV) fraction for predicting the Ki-67 proliferation index in rectal cancer (RC).

**Materials and methods:**

This retrospective study included 117 patients with pathologically confirmed rectal adenocarcinoma who underwent preoperative MRI and DLSCT. Patients were divided into high (Ki-67 > 50%) and low (Ki-67 ≤ 50%) Ki-67 groups based on postoperative immunohistochemistry. Parameters from DKI (mean diffusivity (D_mean_), mean kurtosis [K_mean_]), DWI (apparent diffusion coefficient [ADC]), and ECV fraction were calculated and compared. Diagnostic performance was assessed using the area under the receiver operating characteristic curve (AUC) and decision curve analysis (DCA). Correlations with Ki-67 were evaluated using Spearman’s coefficient.

**Results:**

The high Ki-67 group exhibited significantly higher K_mean_ and ECV fraction, and lower D_mean_ and ADC values (all *p* < 0.05). K_mean_ and ECV showed positive correlations with Ki-67, while ADC showed a negative correlation. ROC analysis demonstrated that ECV fraction provided the optimal diagnostic efficacy (AUC = 0.815), which was significantly better than ADC (AUC = 0.665), D_mean_ (AUC = 0.641), K_mean_ (AUC = 0.721), and combined DKI parameters (AUC = 0.744). DCA confirmed a favorable net benefit for the ECV fraction. Additionally, ECV was associated with key pathological and staging features, like T-stage, N-stage, extramural venous invasion, and other aggressive pathological features.

**Conclusion:**

DLSCT-derived ECV fraction demonstrated superior performance to diffusion-based MRI parameters for predicting the proliferation status of RC, providing a more comprehensive assessment of the tumor microenvironment.

**Key Points:**

- **Question** Noninvasive preoperative biomarkers are lacking to assess Ki-67 proliferation status and guide personalized treatment strategies for patients with rectal cancer.
- **Findings** Dual-layer spectral CT (DLSCT)-derived extracellular volume (ECV) fraction outperformed MRI diffusion parameters for Ki‑67 and correlated with T/N stage, differentiation, and extramural vascular invasion.
- **Critical relevance statement** This study validates Dual-layer spectral CT (DLSCT)-derived extracellular volume (ECV) fraction as a non‑invasive biomarker that outperforms MRI diffusion parameters for Ki‑67 assessment and correlates with tumor aggressiveness, advancing personalized treatment decision‑making in rectal cancer radiology.

**Graphical Abstract:**

**Figure:**
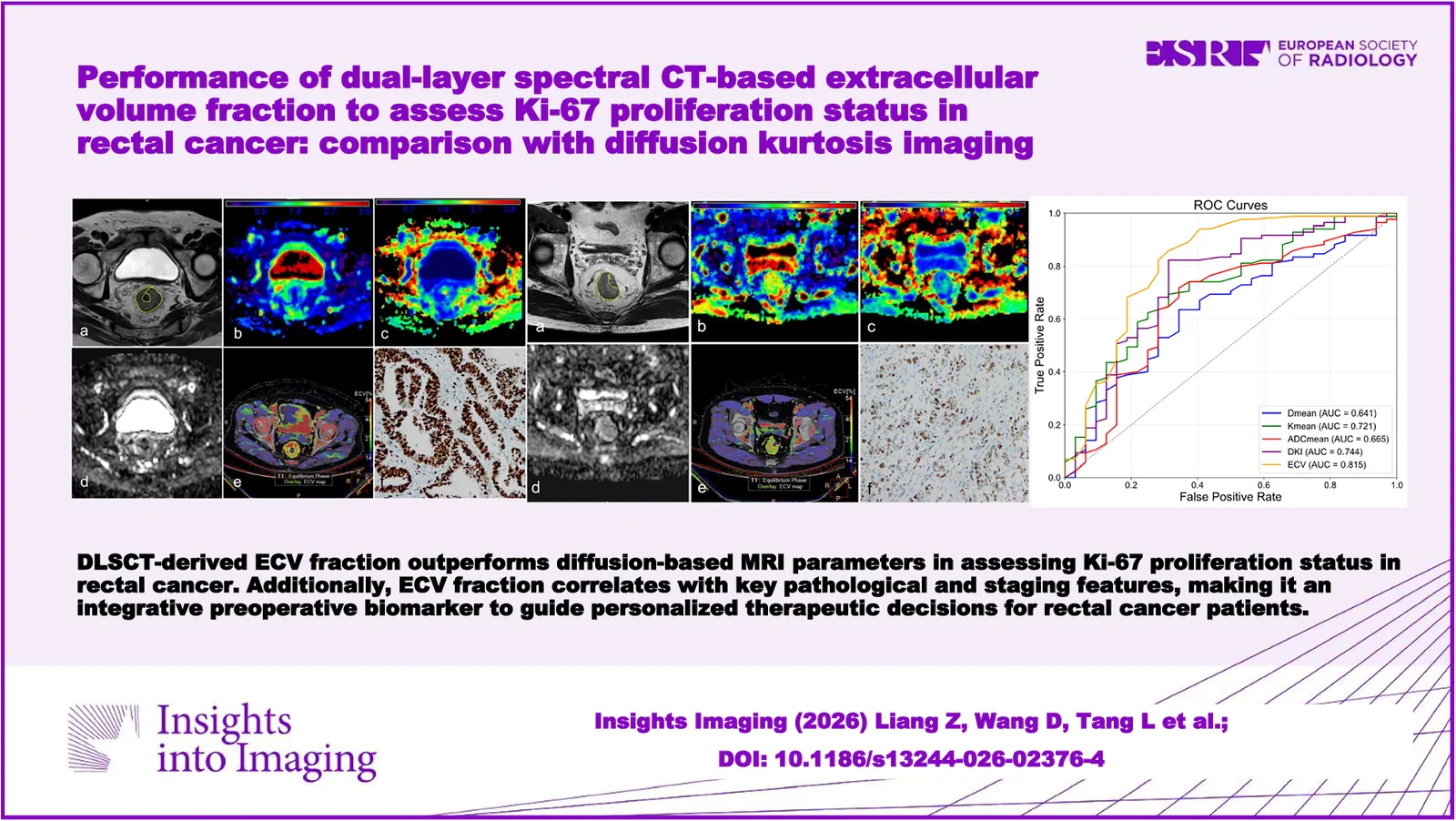

## Introduction

Colorectal cancer is the third most commonly diagnosed cancer and the second leading cause of cancer-related mortality worldwide, with rectal cancer (RC) accounting for 30–35% of cases [1]. Ki-67 is a well-validated nuclear biomarker reflecting tumor cell proliferative activity. In RC, high Ki-67 labeling index (LI) is consistently associated with increased tumor aggressiveness, elevated recurrence risk, and poor overall survival [2–4]. Recent evidence suggests that tumors with high Ki-67 LI may also predict a more favorable response to neoadjuvant chemoradiotherapy and chemotherapy, providing a potential guide for tailoring therapeutic intensity and minimizing treatment related toxicities [5, 6]. Currently, Ki-67 LI assessment relies on postoperative surgical specimens. Although preoperative endoscopic biopsy can also evaluate Ki-67, it carries risks such as perforation, hemorrhage, and even mortality [7, 8]. Therefore, noninvasive and accurate determination of Ki-67 LI is clinically significant for personalized treatment decisions.

Recently, diffusion-weighted imaging (DWI) and diffusion kurtosis imaging (DKI) have been applied in the precise diagnosis and prognostic stratification of RC [9, 10]. For instance, our previous studies combined amide proton transfer imaging with DKI/DWI to evaluate the expression of p53 and Ki-67 [11], as well as other pathological prognostic factors in RC [12]. Our findings revealed that high Ki-67 expression in RC correlates with increased cellular structural complexity and restricted diffusion, reflected in a reduced apparent diffusion coefficient (ADC), decreased mean diffusivity (D_mean_), and elevated mean kurtosis (K_mean_), which are consistent with Yuan et al’s findings [13]. Nevertheless, DWI is limited by the Gaussian diffusion assumption, impairing its accuracy in heterogeneous tumor microenvironments [14]. Additionally, DKI is susceptible to motion artifacts and has relatively low spatial resolution, potentially compromising its reproducibility and clinical utility [15].

The extracellular matrix (ECM) comprises acellular components (collagen, glycoproteins, proteoglycans) that constitute the cellular microenvironment [16]. During tumor progression, ECM plays a critical role in tumor proliferation, differentiation, and metastasis [17]. Extracellular volume (ECV) fraction is defined as the proportion of the extracellular space to the total tissue volume [18]. It can be quantified via imaging-based contrast agent distribution [19]. Alterations in ECV fraction reflect ECM remodeling associated with tumor proliferation, including angiogenesis, fibrosis, and increased deposition of matrix proteins [20]. Therefore, compared to diffusion-based imaging techniques, ECV fraction may provide a more direct and comprehensive assessment of tissue structural changes linked to cellular proliferation. Although ECV fraction has shown value in diagnosing tumors, characterizing pathology, and predicting outcomes in pancreatic and ovarian cancers [21, 22], its potential for assessing proliferative activity in RC remains underexplored.

Although CT cannot supplant high-resolution magnetic resonance imaging (HR-MRI) for local staging, it remains the guideline-recommended standard for systemic assessment [23]. Dual-layer spectral detector CT (DLSCT) can decompose materials from a single contrast-enhanced scan, allowing ECV fraction quantification without additional non-contrast scanning [24], reducing radiation dose and streamlining workflow. This study aims to explore the feasibility of DLSCT-derived ECV fraction as a noninvasive biomarker for Ki-67 expression in RC. We further compared its diagnostic performance with HR-MRI diffusion parameters (ADC and DKI) to identify a robust, clinically applicable imaging marker.

## Materials and methods

### Study population

**Fig. 1:**
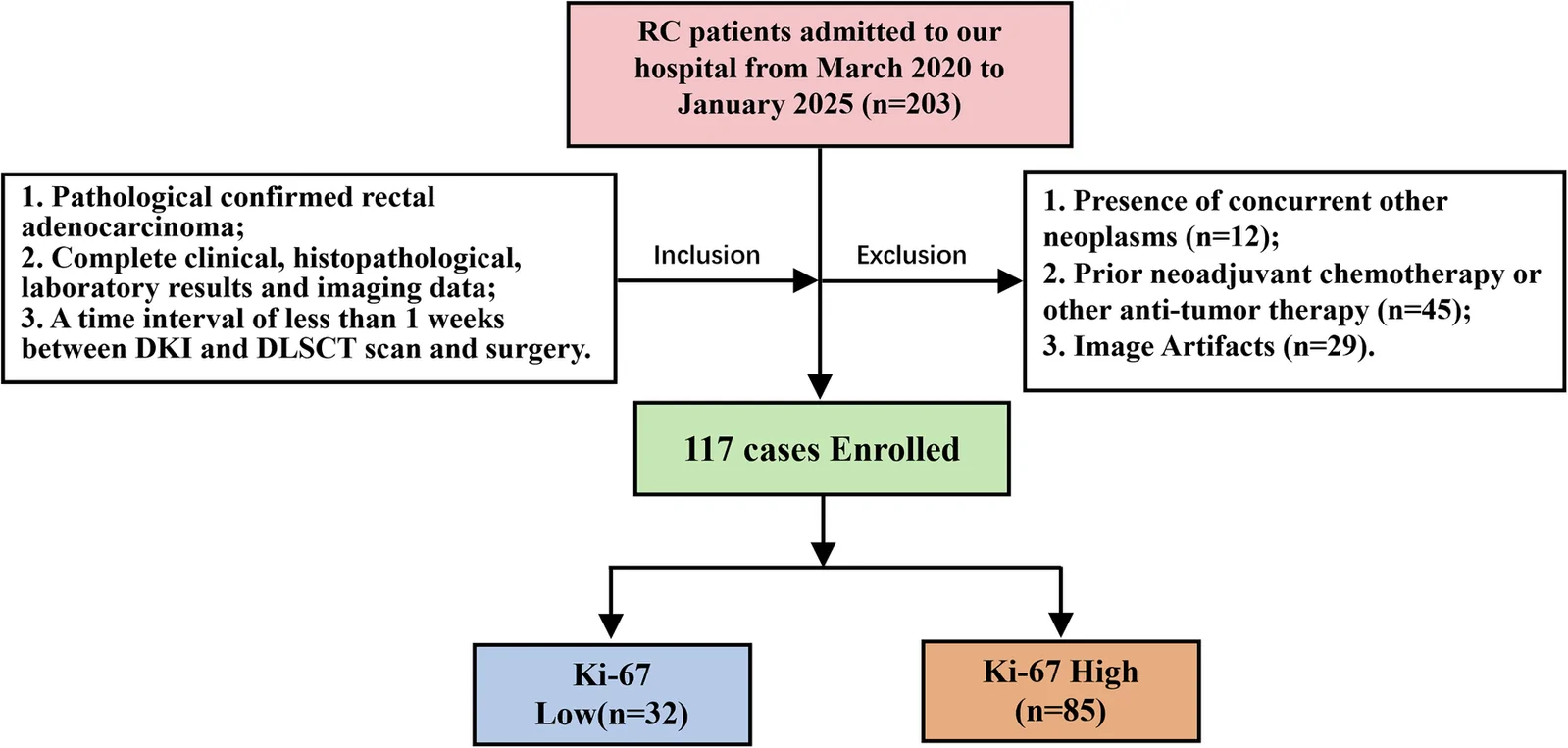
Flowchart of patient selection

This retrospective study was approved by the institutional review board of the hospital, with a waiver of informed consent, under the ethics approval number YE2024-342-01. A total of 117 patients with pathologically confirmed primary RC who underwent surgical resection between March 2020 and January 2025 were enrolled. The inclusion criteria were as follows: (1) Patients with primary rectal adenocarcinoma confirmed by histopathology; (2) Complete clinical data and laboratory results; (3) Completion of both DLSCT and HR-MRI within 1 week prior to surgery; (4) Availability of hematocrit levels within approximately 1 week before CT examination. The exclusion criteria were as follows: (1) Poor image quality or severe artifacts on CT or MR images, including severe metal artifacts from lumbar spinal internal fixation devices (*n* = 25) and motion artifacts caused by bowel peristalsis or poor respiratory coordination (*n* = 4); (2) Presence of concurrent other neoplasms (*n* = 12); (3) Prior antitumor therapy before preoperative imaging (*n* = 45). The process of patient selection is illustrated in Fig. 1.

### Image acquisition

CT examinations were performed using an IQon spectral CT (Philips Healthcare). The scan range extended from the diaphragm to the symphysis pubis. DLSCT scan parameters were as follows: tube voltage, 120 kV; tube current, automated exposure modulation; detection collimation, 64 × 0.625 mm; rotation time, 0.4 s; and helical pitch, 1.1. For contrast-enhanced CT, a non-ionic iodinated contrast agent (Ultravist 370, Bayer Healthcare; 1.5 mL/kg body weight) was intravenously administered via the median cubital vein at a flow rate of 3.5 mL/s, followed by a 50 mL saline flush. Using bolus tracking with a region of interest placed in the abdominal aorta, arterial phase (AP) imaging was triggered at a threshold of 150 Hounsfield units. Venous phase (VP) and delayed phase (DP) images were acquired 40 s and 100 s after AP initiation, respectively.

Rectal MRI was conducted using 3.0-T scanners (Philips Ingenia, Philips Healthcare) with a 32-channel phased-array body coil. Patients underwent rectal cleansing before MR scanning. To minimize bowel movement, 5 mg raceanisodamine hydrochloride was administered via intramuscular injection 30 min before the MRI examination [25]. The standard staging MRI protocol consisted of high-resolution T2-weighted fast spin-echo sequences without fat saturation in three directions. The oblique axial and oblique coronal planes were perpendicular and parallel to the long axis of the tumor as identified on sagittal MRI. DKI (b = 0, 600, 1000, 2000 s/mm2) and conventional DWI (b = 0, 1000 s/mm2) sequences were performed in the transverse plane using a single-shot echo-planar imaging sequence with identical parameters. For DKI, the parallel imaging acceleration factor was 2, and the number of gradient directions was 32 for each non-zero b value. All detailed sequence parameters are listed in Table 1.

**Table 1:** Image acquisition parameters for DLSCT and MRI

Parameter	IQon spectral CT	3.0-T MR		
Sequence		TSE	DWI	DKI
Flip angle (degree)	—	90	90	90
TR	—	3900 ms	3000 ms	3000 ms
TE	—	100 ms	72 ms	99 ms
Section thickness	1 mm	3 mm	3 mm	3 mm
Intersection gap	—	0	0	0
FOV	-	200 × 200 mm	222 × 222 mm	222 × 222 mm
Matrix	512 × 512	288 × 288	84 × 82	84 × 82
b values (s/mm²)	—	—	0, 1000	0, 600, 1000, 2000

*DKI* diffusion kurtosis imaging, *DLSCT* dual-layer spectral computed tomography, *DWI* diffusion-weighted imaging, *EPI* echo-planar imaging, *FOV* field of view, *TE* echo time, *TR* repetition time, *TSE* turbo spin echo

### Image processing and analysis

All image analyses were performed independently by two radiologists (each with over 8 years of experience in gastrointestinal oncology imaging) under a double-blind protocol.

#### DLSCT image analysis and ECV calculation

DLSCT image reconstruction was performed using post-processing software (IntelliSpace Portal, Version 10.0, Philips Healthcare). Spectral imaging data were postprocessed to generate iodine maps and virtual mono-energetic images (VMI). The two radiologists evaluated the data according to the following steps: First, 40-keV VMI images in the AP and VP were used to identify tumor margins. Second, as shown in Supplementary Fig. 1, on DP-iodine maps, a freehand ROI was manually drawn along the tumor boundary to measure tumor iodine concentration (IC), carefully excluding fat, hemorrhage, vessels, and calcifications. Third, IC values of the common or external iliac artery were also measured. Fourth, ECV fraction for the tumor was calculated using the formula: ECV (%) = (1 − hematocrit) × (IC__tumor_ / IC__artery_) × 100%. The final ECV value used for subsequent statistical analysis was the average of the two ECV values calculated by the two radiologists.

#### MR image analysis

The Diffusional Kurtosis Estimator software (Version 2.6.0) was used to analyze both DWI and DKI data. Following the analytical approaches described in previous studies [26, 27], a Gaussian filter was initially applied to all diffusion-weighted images. For the DWI dataset, a two-variable linear least-squares method was employed to generate pixel-wise ADC maps based on a monoexponential model, using the equation: ln(S) = ln(S₀) − b · ADC, where S₀ and ADC are the fitting variables. In this equation, S represents the measured signal intensity for a given diffusion-weighting value b, and S₀ is the signal ntensity when b = 0. For the DKI dataset, a three-variable linear least-squares method was applied to compute pixel-wise kurtosis and diffusivity maps according to the DKI model. The model follows the equation: ln(S) = ln(S₀) − b · D + (1/6) · b² · D² · K, with S₀, D, and K as fitting variables, where S is the signal at a specific b value, S₀ is the baseline signal without diffusion gradient, K is kurtosis, and D is diffusivity.

ROI delineation for the MR image was performed using the segmentation module in the 3D-Slicer software (Version 4.10.2). Axial T2-weighted fat-suppressed images served as anatomical reference. Two radiologists manually determined the entire tumor volume by manually drawing ROIs along the border of the tumor on each consecutive ADC map covering the whole lesion, and excluded the fat, hemorrhage, vessels, and calcifications by referring to the T2-weighted images. ROIs were drawn with the same criteria on D_mean_ maps and were later copied to K_mean_ maps. These volumetric ROIs were then propagated to the co-registered parametric maps (ADC, D_mean_, K_mean_) to compute the mean value of each parameter within the entire tumor volume [28]. This process was performed independently by each radiologist, and the final ADC, D_mean_, and K_mean_ values used for statistical analysis were the average of their two measurements.

### Analyses of Ki-67 expression

All patients underwent surgical resection within 1 week after completing DLSCT and MRI examinations. Ki-67 expression was assessed using immunohistochemistry with the mouse anti-human monoclonal antibody MIB-1 (Dako). For each case, five hotspot regions exhibiting high densities of positively stained cells were identified at ×400 magnification. Within each hotspot, 100 tumor cells were counted to determine the percentage of positively stained nuclei, reflecting the Ki-67 expression level. Patients exhibiting weakly positive (+, < 10%) or moderately positive (++, 10–50%) Ki-67 were classified into the low Ki-67 group, whereas those with strongly positive expression (+++, > 50%) were classified into the high Ki-67 group [29, 30].

### Statistical analysis

The interobserver and intra-observer agreement for the quantitative measurements was assessed using the intraclass correlation coefficient (ICC). Specifically, a subset of 30 patients was randomly selected from the full cohort; for these cases, measurements were performed independently by two radiologists to assess interobserver agreement and repeated by one radiologist after a 4-week interval to assess intra-observer agreement. This analysis specifically evaluated the consistency between the two radiologists for the following parameters: the IC__tumor_, IC__artery_, and ECV fraction from DLSCT, and the ADC, D_mean_, and K_mean_ values from MRI. The ICC values were interpreted as follows: < 0.40, poor; 0.40–0.59, fair; 0.60–0.74, good; and ≥ 0.75, excellent.

To compare the differences in each parameter between the high and low Ki-67 groups, continuous variables were compared using the Student’s *t*-test or Mann–Whitney U test, while categorical variables were compared using the chi-square test or Fisher’s exact test, and for comparisons among more than two groups, one-way analysis of variance (ANOVA) or Kruskal–Wallis H test was used. The Spearman correlation analysis was employed to analyze relationships between imaging parameters (K_mean_, D_mean_, ADC_mean_, ECV fraction) and Ki-67 expression. The diagnostic performance of each parameter was assessed using the area under the receiver operating characteristic curve (AUC). Furthermore, in order to evaluate the predictive accuracy and clinical usefulness, decision curve analysis (DCA) was conducted. All statistical analyses were conducted using Python (version 3.9.5) and SPSS (version 26.0, IBM Corp.), with a two-sided *p*-value < 0.05 considered statistically significant.

## Results

### Patient characteristics

A total of 117 patients with pathologically confirmed RC were enrolled in this study, comprising 82 men and 35 women. Based on Ki-67 expression, 85 patients were classified into the high Ki‑67 group and 32 into the low Ki‑67 group. Compared with the low Ki‑67 group, the high Ki‑67 group was significantly associated with elevated CA19‑9 (*p* = 0.001), more advanced pT stage (*p* < 0.001), and higher HER2 positivity (*p* = 0.038). Detailed demographic and clinicopathological characteristics of the patients are presented in Table 2.

**Table 2:** Clinical and pathological characteristics of enrolled patients

Characteristics	Total	Low Ki-67	High Ki-67	*p*-value
No. of patients	117	32	85	
Age, median (IQR)	62.0 (18.0)	65.0 (16.0)	65.0 (21.0)	1.000
Gender, no. (%)				0.518
Male	82 (70.1%)	21 (65.6%)	61 (71.8%)	
Female	35 (29.9%)	11 (34.4%)	24 (28.2%)	
CEA, no. (%)				0.510
≤ 5 ng/mL	60 (51.3%)	18 (56.2%)	42 (49.4%)	
> 5 ng/mL	57 (48.7%)	14 (43.8%)	43 (50.6%)	
CA19-9, no. (%)				0.001
< 30 U/mL	75 (64.1%)	28 (87.5%)	47 (55.3%)	
≥ 30 U/mL	42 (35.9%)	4 (12.5%)	38 (44.7%)	
pT stage, no. (%)				< 0.001
T1	6 (5.1%)	0 (0.0%)	6 (7.1%)	
T2	33 (28.2%)	18 (56.2%)	15 (17.6%)	
T3	68 (58.1%)	13 (40.6%)	55 (64.7%)	
T4	10 (8.6%)	1 (3.1%)	9 (10.6%)	
pN stage, no. (%)				0.223
N0	66 (56.4%)	22 (68.8%)	44 (51.8%)	
N1	37 (31.6%)	8 (25.0%)	29 (34.1%)	
N2	14 (12.0%)	2 (6.2%)	12 (14.1%)	
Differentiation, no. (%)				0.054
Well	8 (6.8%)	4 (12.5%)	4 (4.7%)	
Moderate	69 (59.0%)	22 (68.8%)	47 (55.3%)	
Poor	40 (34.2%)	6 (18.8%)	34 (40.0%)	
MSI status, no. (%)				1.000
MSS	103 (88.0%)	28 (87.5%)	75 (88.2%)	
MSI-H	14 (12.0%)	4 (12.5%)	10 (11.8%)	
HER2 status, no. (%)				0.038
Negative	100 (85.5%)	31 (96.9%)	69 (81.2%)	
Positive	17 (14.5%)	1 (3.1%)	16 (18.8%)	
EMVI, no. (%)				0.879
Negative	94 (80.3%)	26 (81.2%)	68 (80.0%)	
Positive	23 (19.7%)	6 (18.8%)	17 (20.0%)	

*CA19-9* carbohydrate antigen 19-9, *CEA* carcinoembryonic antigen, *EMVI* extramural venous invasion, *HER2* human epidermal growth factor receptor 2, *IQR* interquartile range, *MSI* microsatellite instability, *MSI-H* microsatellite instability-high, *MSS* microsatellite stable

### Measurement time

The measurement time for CT and MR parameters was approximately 8–13 min per case, depending on tumor size and complexity.

### Consistency evaluation

The ICCs for IC__tumor_, IC__artery_, ECV fraction, ADC, K_mean_, and D_mean_, as measured by two independent observers, ranged from 0.844 to 0.986 for interobserver agreement and from 0.831 to 0.973 for intra-observer agreement, both indicating excellent interobserver  and intra-observer agreement. The specific ICC values of each parameter are detailed in Supplementary Table 1.

### Parameter comparison

The ECV fraction exhibited significant differences in relation to the pT stage (*H* = 22.285, *p* < 0.001) and pN stage (*H* = 23.599, *p* < 0.001). It was also significantly higher in patients with extramural vascular invasion (EMVI) than in those without (40.274 (11.273) vs. 36.208 (6.284); *Z* = −2.372, *p* = 0.018).

Regarding tumor differentiation, significant differences were found in D_mean_ (*H* = 8.287, *p* = 0.016), K_mean_ (*H* = 6.921, *p* = 0.031), and ECV (*H* = 30.953, *p* < 0.001).

**Fig. 2:**
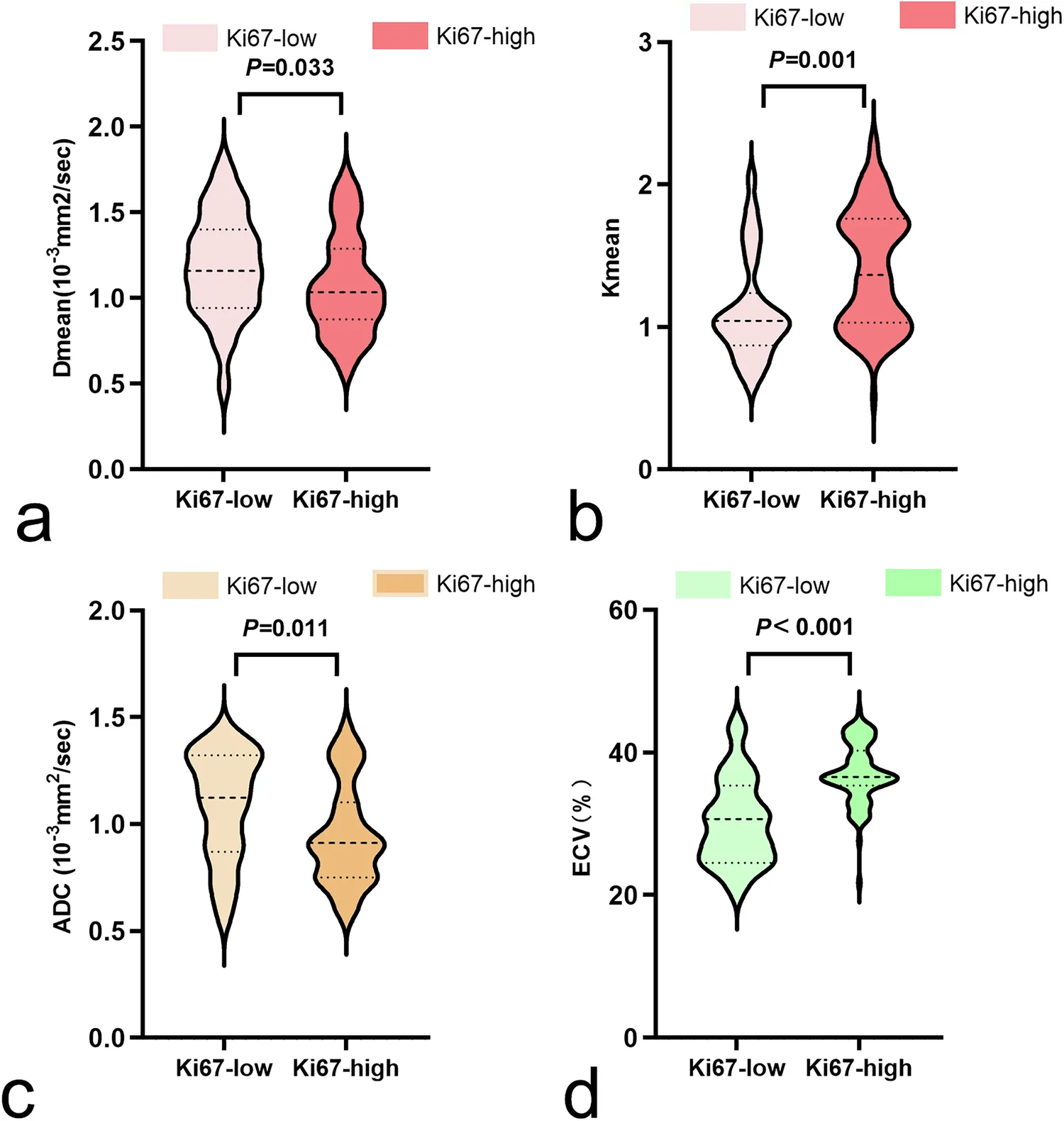
Violin plots showing the distributions of D_mean_ (**a**), K_mean_ (**b**), ADC_mean_ (**c**) and extracellular volume (ECV) (**d**) fraction between Ki-67-low and Ki-67-high rectal cancer (RC). Ki-67-high RC exhibited significantly higher K_mean_ and ECV fraction compared to Ki-67-low RC, along with lower D_mean_ and ADC_mean_ values (all *p* < 0.05)

For proliferation status as determined by Ki-67 LI, the high Ki-67 group had significantly higher K_mean_ (1.367 (0.731) vs. 1.043 (0.366); *Z* = −3.447, *p* = 0.001) and ECV fraction (36.575 (4.900) vs. 30.670 (10.835); *Z* = −5.24, *p* < 0.001), and lower D_mean_ (1.034 (0.414) vs. 1.160 (0.459); *Z* = −2.131, *p* = 0.033) and ADC_mean_ (0.913 (0.351) vs. 1.123 (0.453); *Z* = −2.53, *p* = 0.011) compared to the low Ki-67 group (Fig. 2). The detailed results for all parameters are summarized in Table 3.

**Table 3:** Comparison of mean diffusivity, kurtosis, ADC, and ECV between different pathological statuses

	D_mean_ (10^–^^3^ mm^2^/s)	K_mean_	ADC_mean_ (10^–^^3^ mm^2^/s)	ECV (%)
pT stage				
T1 (*n* = 6)	1.112 (0.430)	1.064 (0.420)	1.203 (0.573)	36.392 (2.916)
T2 (*n* = 33)	1.053 (0.460)	1.062 (0.868)	0.954 (0.505)	32.227 (11.171)
T3 (*n* = 68)	1.079 (0.409)	1.273 (0.664)	0.914 (0.398)	36.344 (5.964)
T4 (*n* = 10)	0.934 (0.925)	1.680 (0.757)	0.915 (0.446)	43.252 (1.444)
*H* value	0.329	4.805	1.904	22.285
*p*-value	0.956	0.187	0.593	< 0.001
pN stage				
N0 (*n* = 66)	1.068 (0.407)	1.102 (0.779)	0.933 (0.494)	35.375 (5.918)
N1 (*n* = 37)	1.083 (0.092)	1.239 (0.653)	0.916 (0.488)	36.575 (9.581)
N2 (*n* = 14)	1.017 (0.550)	1.657 (0.549)	0.914 (0.288)	38.954 (4.298)
*H* value	0.391	4.054	0.364	23.599
*p*-value	0.822	0.132	0.834	< 0.001
Differentiation				
Well (*n* = 8)	1.302 (0.441)	1.049 (0.328)	1.117 (0.704)	32.227 (6.502)
Moderate (*n* = 69)	1.090 (0.401)	1.093 (0.765)	0.916 (0.528)	35.374 (6.486)
Poor (*n* = 40)	0.971 (0.376)	1.425 (0.639)	0.924 (0.246)	41.903 (6.668)
*H* value	8.287	6.921	0.275	30.953
*p*-value	0.016	0.031	0.871	< 0.001
EMVI				
Negative (*n* = 94)	1.086 (0.416)	1.172 (0.717)	0.919 (0.493)	36.208 (6.284)
Positive (*n* = 23)	0.995 (0.430)	1.452 (0.765)	0.917 (0.311)	40.274 (11.273)
*Z* value	−1.410	−0.686	−0.127	−2.372
*p*-value	0.159	0.493	0.899	0.018
Ki-67 status				
Low (*n* = 32)	1.160 (0.459)	1.043 (0.366)	1.123 (0.453)	30.670 (10.835)
High (*n* = 85)	1.034 (0.414)	1.367 (0.731)	0.913 (0.351)	36.575 (4.900)
*Z* value	−2.131	−3.447	−2.530	−5.240
*p*-value	0.033	0.001	0.011	< 0.001

Continuous data were assessed for normality using the Shapiro–Wilk test. Non-normally distributed variables are presented as median (interquartile range). Grading of tumors was based on the WHO grading criteria

*ADC* apparent diffusion coefficient, *D*_*mean*_ mean diffusivity, *ECV* extracellular volume fraction, *EMVI* extramural venous invasion, *K*_*mean*_ mean kurtosis

### Diagnostic performance

**Fig. 3:**
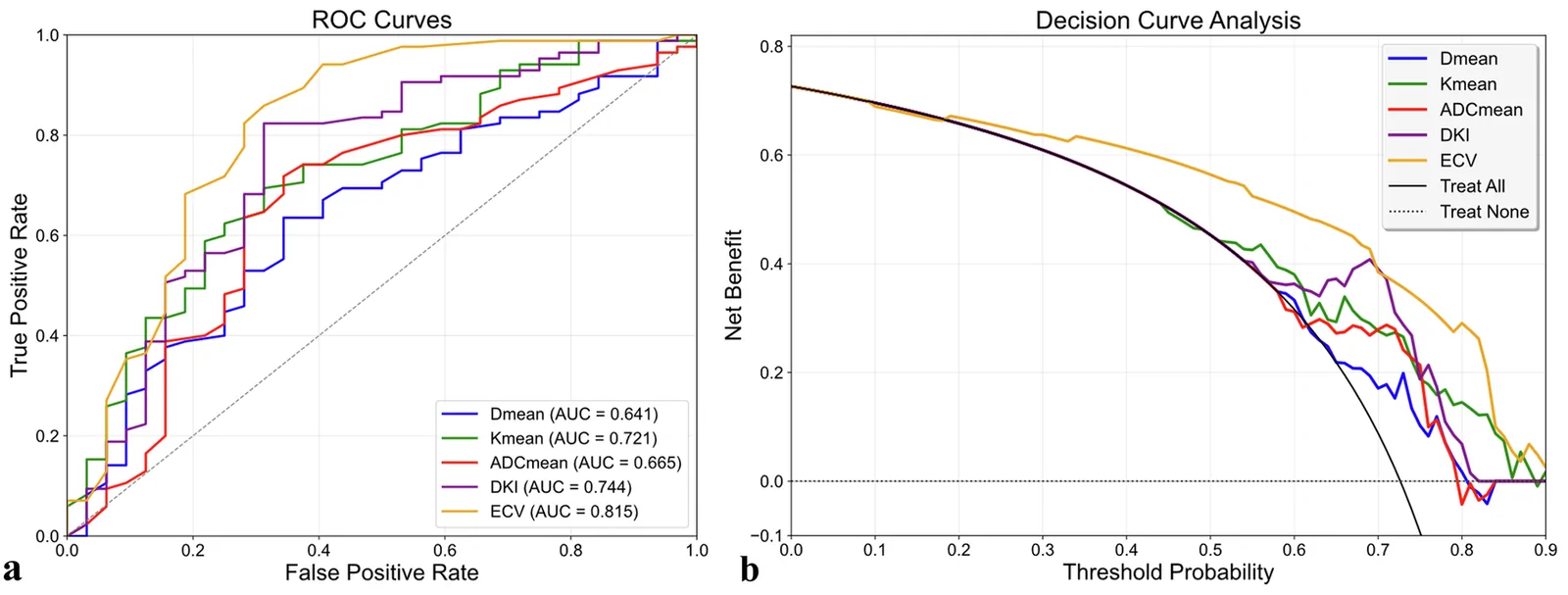
**a** Receiver operating characteristic analysis of D_mean_, K_mean_, ADC_mean_, DKI model, and extracellular volume (ECV) fraction for discriminating Ki-67 expression status of rectal cancer (RC). **b** Decision curve analysis (DCA) curves showed that the ECV fraction was more effective in predicting Ki-67 expression status, offering better net benefits for threshold probabilities from 0.00 to 0.90

ECV fraction demonstrated the highest diagnostic performance for distinguishing between high and low Ki-67 groups (Fig. 3a), with an AUC of 0.815 (95% CI: 0.714–0.915), which was superior to that of all other parameters. The optimal cutoff value, determined by maximizing Youden’s index, was 32.227%, yielding a sensitivity of 68.75% and a specificity of 85.88%. To evaluate the influence of imbalanced sample distribution on model stability, bootstrapping with 1000 resamplings was performed for AUC estimation. The bootstrapped AUC of ECV fraction for predicting high Ki‑67 status ranged from 0.710 to 0.911, indicating consistent and robust diagnostic performance.

In comparison, the diagnostic metrics for other parameters were as follows: DKI (AUC = 0.744, 95% CI: 0.635–0.853; sensitivity: 68.75%; specificity: 82.35%), K_mean_ (AUC = 0.721, 95% CI: 0.617–0.825; sensitivity: 68.75%; specificity: 69.41%), ADC_mean_ (AUC = 0.665, 95% CI: 0.547–0.782; sensitivity: 71.76%; specificity: 65.62%), and D_mean_ (AUC = 0.641, 95% CI: 0.530–0.754; sensitivity: 63.53%; specificity: 65.62%). Detailed results are summarized in Table 4. Furthermore, DCA curves confirmed the superior clinical utility of ECV fraction, which provided a higher net benefit across threshold probabilities from 0.00 to 0.90 compared to the other parameters (Fig. 3b).

**Table 4:** ROC analysis of mean diffusivity, kurtosis, ADC, and ECV fraction in differentiating Ki-67 low from high status

	AUC (95% CI)	Accuracy	Sensitivity	Specificity	PPV	NPV	*p*-value
D_mean_	0.641 (0.530–0.754)	64.10%	63.53%	65.62%	83.08%	40.38%	0.013
K_mean_	0.721 (0.617–0.825)	69.23%	68.75%	69.41%	45.83%	85.51%	< 0.001
ADC_mean_	0.665 (0.547–0.782)	69.09%	71.76%	65.62%	84.72%	46.67%	0.006
DKI	0.744 (0.635–0.853)	78.63%	68.75%	82.35%	59.46%	87.50%	< 0.001
ECV	0.815 (0.714–0.915)	81.20%	68.75%	85.88%	64.71%	87.95%	< 0.001

*ADC* apparent diffusion coefficient, *AUC* area under the curve, *DKI* diffusion kurtosis imaging, *D*_*mean*_ mean diffusivity, *ECV* extracellular volume fraction, *K*_*mean*_ mean kurtosis, *NPV* negative predictive value, *PPV* positive predictive value, *ROC* receiver operating characteristic

### Analysis of correlation

**Fig. 4:**
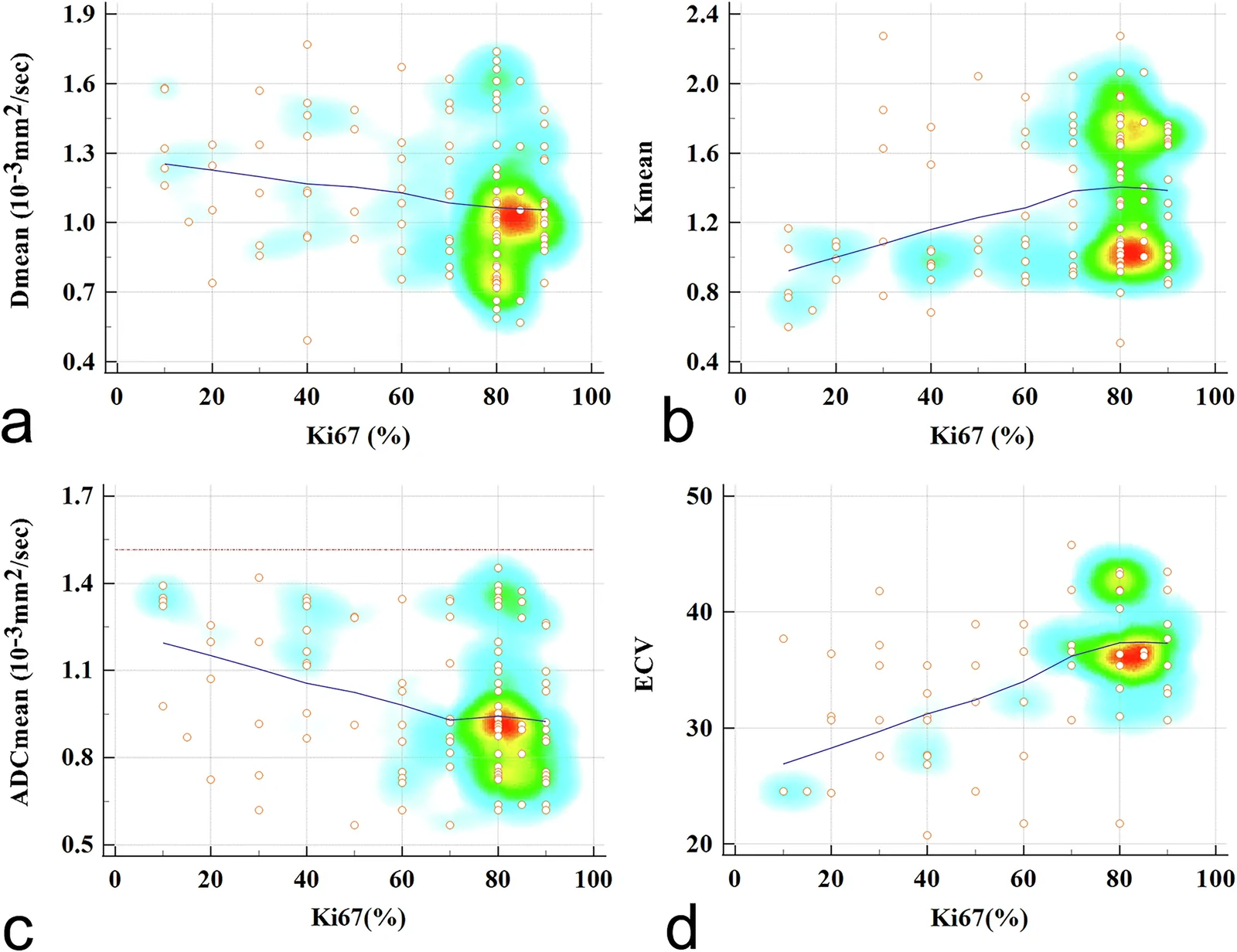
Spearman correlation of D_mean_ (**a** *r* = −0.159, *p* = 0.086), K_mean_ (**b** *r* = 0.241, *p* = 0.009), ADC_mean_ (**c** *r* = −0.222, *p* = 0.016), and extracellular volume (ECV) fraction (**d** *r* = 0.396, *p* < 0.001) with Ki-67 labeling index

Spearman correlation analysis was performed to assess the relationships of K_mean_, D_mean_, ADC_mean_, and ECV fraction with the Ki-67 LI (Fig. 4). The analysis revealed significant positive correlations for both K_mean_ and ECV fraction with the Ki-67 LI (*p* < 0.05). A significant negative correlation was observed between ADC_mean_ and Ki-67 LI (*p* = 0.016). Although D_mean_ also showed a negative trend with Ki-67 LI, this correlation did not reach statistical significance (*p* > 0.05). Detailed results are presented in Table 5.

**Table 5:** Spearman correlation coefficients between mean diffusivity, kurtosis, ADC, ECV, and Ki-67 labeling index

Ki-67 index	D_mean_ (10^–^^3^ mm^2^/s)	K_mean_	ADC_mean_ (10^–^^3^ mm^2^/s)	ECV (%)
Spearman correlation coefficient	−0.159	0.241	−0.222	0.396
*p*-value	0.086	0.009	0.016	< 0.001

*ADC* apparent diffusion coefficient, *D*_*mean*_ mean diffusivity, *ECV* extracellular volume fraction, *K*_*mean*_ mean kurtosis

**Fig. 5:**
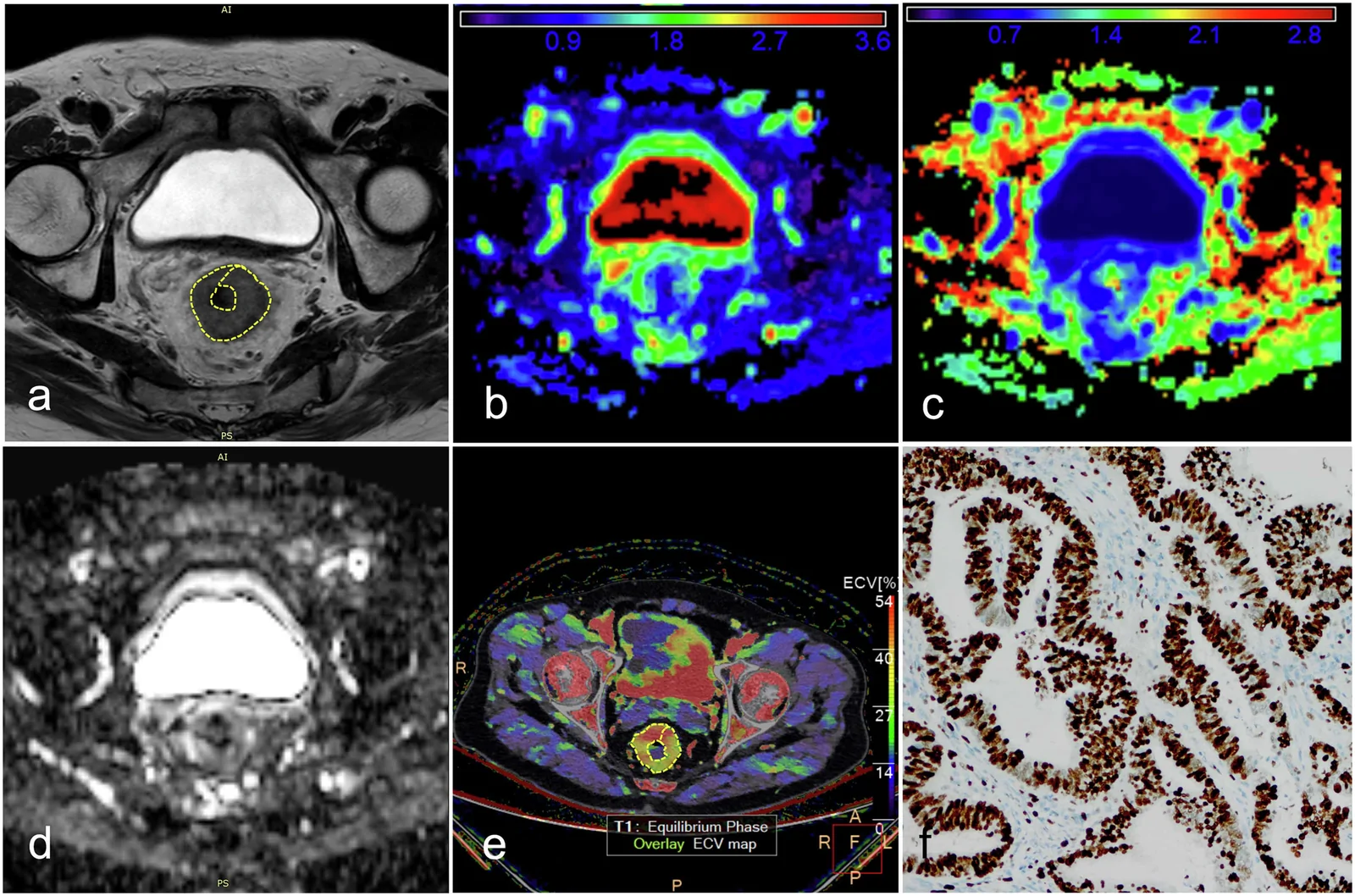
Images in a 57-year-old male with grade 2 (according to WHO criteria) rectal adenocarcinoma with high Ki-67 expression. **a** Axial T2-weighted MRI shows a slightly low-signal-intensity rectal mass. **b** The diffusion map shows the lesion with a D_mean_ value of 1.076 × 10^–^^3^ mm^2^/s. **c** The kurtosis map demonstrates the lesion with a K_mean_ value of 1.167. **d** The apparent diffusion coefficient map shows a low-intensity tumor, with an ADC_mean_ value of 0.965 × 10^–^^3^ mm^2^/s. **e** The extracellular volume (ECV) map was reconstructed using hematocrit, and the iodine density value in the delayed phase demonstrated an ECV fraction of 40.9%. **f** Immunohistochemical staining shows that 90% of cells are Ki-67-positive (×100)

**Fig. 6:**
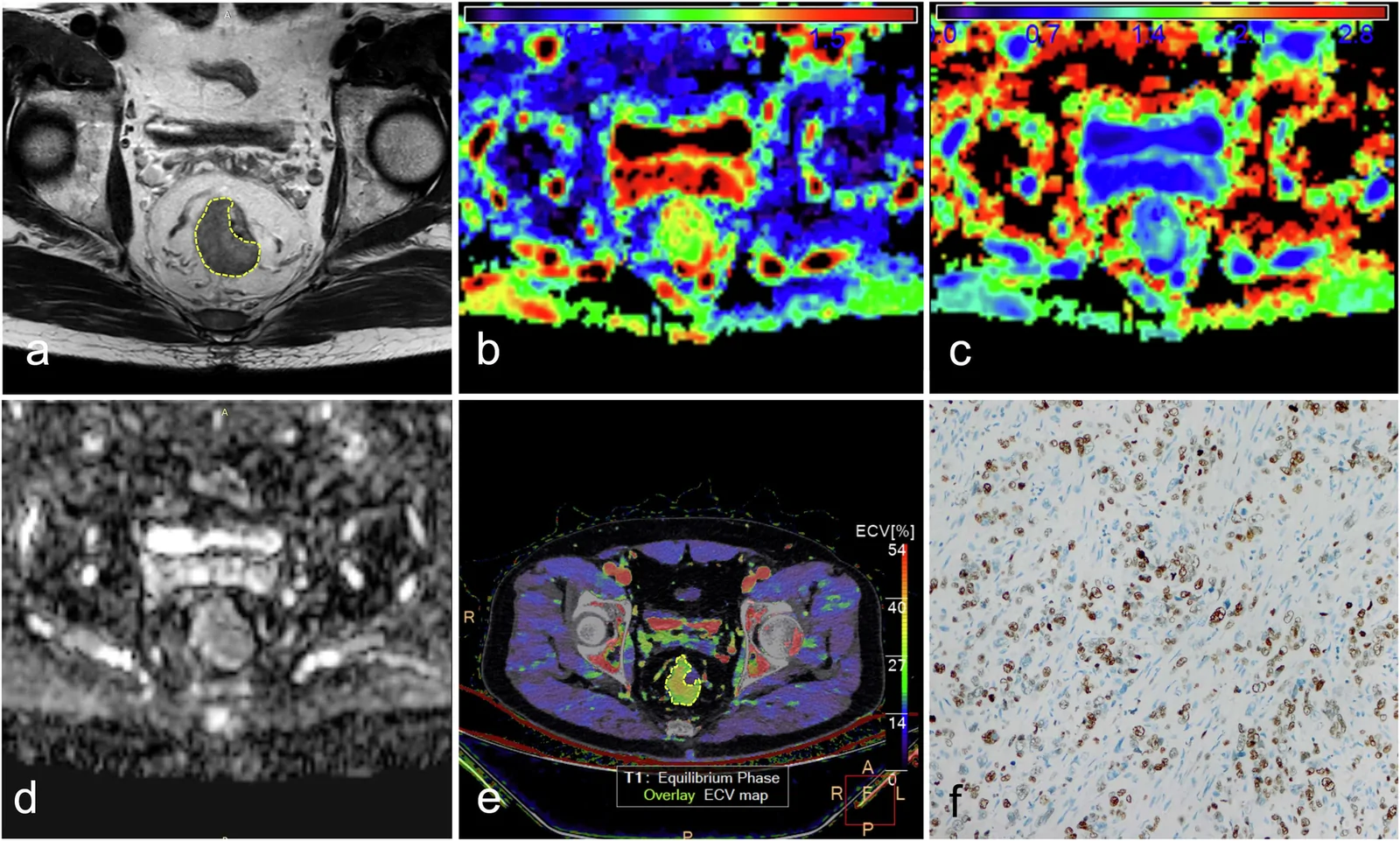
Images in a 63-year-old male with grade 3 (according to WHO criteria) rectal adenocarcinoma with low Ki-67 expression. **a** Axial T2-weighted MRI shows a slightly low-signal-intensity rectal mass. **b** The diffusion map shows the lesion with a D_mean_ value of 1.505 × 10^–^^3^ mm^2^/s. **c** The kurtosis map demonstrates the lesion with a K_mean_ value of 1.013. **d** The apparent diffusion coefficient map shows a low-intensity tumor, with an ADC_mean_ value of 1.291 × 10^–^^3^ mm^2^/s. **e** Extracellular volume (ECV) maps demonstrated the fraction of 30.1%. **f** Immunohistochemical staining shows low Ki-67 expression at 30% (×100)

Figures 5 and 6 showcase two paradigmatic cases of rectal adenocarcinoma with contrasting Ki-67 expression (high in Fig. 5 and low in Fig. 6), presenting corresponding axial T2-weighted MRI, quantitative parameter maps (D_mean_, K_mean_, ADC_mean_, ECV fraction), and confirmatory immunohistochemical staining.

## Discussion

Our results demonstrated that ECV fraction was correlated positively with Ki-67 and achieved the optimal diagnostic performance in distinguishing high and low Ki-67 groups, outperforming ADC_mean_, D_mean_, K_mean_, and combined DKI parameters. Additionally, ECV fraction was significantly associated with multiple prognostic features, including T/N stage, pathological differentiation grade, and EMVI, whereas DKI parameters only correlated with pathological differentiation grade. These findings confirm DLSCT-derived ECV fraction as a valuable noninvasive biomarker for assessing RC proliferative activity.

The superior diagnostic efficacy of ECV fraction over diffusion-based MRI parameters originates from the distinct biological information captured by each modality. Diffusion-based MRI techniques (DWI and DKI) primarily reflect water molecule movement, indirectly influenced by cellular density and tissue complexity [31, 32]. High Ki-67 expression correlates with increased cellular density and restricted diffusion, manifested as decreased ADC_mean_/D_mean_ and increased K_mean_ [13, 33], but these parameters adopt a cell-centric perspective, failing to fully capture tumor microenvironment remodeling during proliferation. In contrast, ECV fraction directly quantifies the extracellular space proportion by measuring contrast agent distribution at equilibrium [34, 35]. The ECM regulates tumor proliferation, invasion, and metastasis [36, 37]. During active proliferation (high Ki-67 LI), tumor and stromal cells secrete ECM components, leading to stromal fibrosis and extracellular space expansion [38]. Concurrently, proliferative tumors induce immature, permeable vascular networks that enhance contrast extravasation [39]. These processes collectively elevate ECV fraction, enabling it to directly reflect ECM structural and functional alterations linked to proliferation. This unique pathophysiological basis explains why ECV fraction demonstrates a stronger correlation with Ki-67 LI and superior diagnostic performance in distinguishing proliferation status.

Prior ECV fraction research has focused on pancreatic and ovarian cancers [21, 22], with limited exploration in RC. Li et al [40] reported that conventional CT-derived ECV fraction could predict pathological tumor grade in RC with high accuracy (AUC = 0.892), which is correlated with Ki-67 status. However, their study focused on tumor grading rather than direct correlation with Ki-67 LI, and the use of conventional CT required separate non-contrast and DP scans, introducing the risk of registration artifacts. Our study builds on this work by directly investigating the correlation between ECV fraction and Ki-67 LI, utilizing DLSCT-derived iodine density maps from the DP for accurate ECV measurement, and expanding the analysis to T/N stage and EMVI to demonstrate ECV fraction’s integrative value beyond mere proliferation assessment. In terms of diffusion imaging, numerous studies validate diffusion parameters for Ki-67 evaluation in RC [9, 10], and our findings are consistent with these studies, confirming that diffusion parameters can distinguish between high and low Ki-67 groups to a certain extent. To our knowledge, this study is the first to conduct a direct and comprehensive comparison between DLSCT-derived ECV fraction and diffusion-based MRI parameters in RC, clearly demonstrating the superior diagnostic efficacy of ECV fraction. While HR-MRI remains indispensable for local staging, ECV fraction complements it by providing unique proliferation and microenvironment information, particularly in patients unable to undergo MRI.

An important insight from this study is that ECV fraction is not only associated with Ki-67 expression but also closely linked to multiple aggressive pathological features of RC. Specifically, ECV fraction differs significantly among different T/N stages, with higher values observed in advanced T4 and N2 stages compared to early-stage tumors. Additionally, ECV fraction is significantly higher in poorly differentiated tumors and those with EMVI compared to well/moderately differentiated tumors and those without EMVI. In contrast, DKI parameters (D_mean_ and K_mean_) only show significant differences with pathological differentiation and not with T/N stage or EMVI. This distinction highlights the unique ability of ECV fraction to capture the comprehensive biological behavior of tumors. T/N stage reflects the extent of tumor invasion and lymph node metastasis, EMVI is an established independent prognostic factor for RC recurrence and overall survival, and pathological differentiation is closely related to tumor aggressiveness and treatment response [41]. The associations between ECV fraction and these key features suggest that ECV fraction can integrate information on tumor proliferation, invasion, and metastatic potential, providing a more holistic understanding of tumor biology preoperatively.

The 2025 ESMO Clinical Practice Guidelines for RC recommend HR-MRI for local staging but lack noninvasive biomarkers for proliferative activity [42], limiting personalized neoadjuvant therapy. Tumors with high Ki-67 LI are more responsive to intensified neoadjuvant therapy, while low Ki-67 LI tumors may not benefit and risk overtreatment [43]. The optimal ECV fraction cutoff (32.227%) identified in this study offers a solution: preoperative ECV fraction above the cutoff may indicate high proliferative activity and aggressive biology, warranting intensified therapy, while values below the cutoff may suit standard regimens or watch-and-wait strategies. Additionally, ECV fraction’s association with T/N stage and EMVI enables comprehensive preoperative risk stratification, aiding prognosis prediction and follow-up planning.

This study has several limitations. First, the retrospective single-center design, limited sample size (*n* = 117), and imbalanced Ki-67 groups (85 vs. 32 patients) may introduce selection bias and compromise generalizability. Bootstrapping analysis supported the robustness of ECV fraction performance; however, formal data balancing techniques (e.g., SMOTE) were not applied. Future multi-center, prospective studies with larger and balanced cohorts are needed to validate the ECV cutoff and confirm prognostic value. Second, 29 patients were excluded due to image artifacts, mostly metal artifacts from spinal implants (25/29) and motion artifacts (4/29). These artifacts may affect ECV and DKI quantification. In future studies, advanced techniques—such as metal artifact reduction algorithms, accelerated MRI sequences (e.g., compressed sensing), enhanced breath-hold training, and stricter bowel preparation—can be used to reduce the impact of artifacts. Third, manual ROI delineation may cause interobserver variability; multi-dimensional imaging information (e.g., texture features, radiomics) was not integrated, and the lack of long-term follow-up data precluded survival analysis. Therefore, future efforts should incorporate AI-based auto-segmentation to improve reproducibility, leverage multi-parametric imaging data, and conduct longitudinal outcome studies to confirm the prognostic relevance of the ECV fraction.

## Conclusion

In conclusion, DLSCT-derived ECV fraction outperforms diffusion-based MRI parameters in assessing Ki-67 proliferation status in RC. Additionally, ECV fraction correlates with key pathological and staging features, making it an integrative preoperative biomarker to guide personalized therapeutic decisions for RC patients.

## Supplementary information


Supplementary information


## Data Availability

The data analyzed during the current study are not publicly available but are available from the corresponding author on reasonable request.

## Abbreviations

ADC: Apparent diffusion coefficient
AP: Arterial phase
AUC: Area under the receiver operating characteristic curve
CI: Confidence interval
CT: Computed tomography
DCA: Decision curve analysis
DKI: Diffusion kurtosis imaging
DLSCT: Dual-layer spectral detector computed tomography
Dmean: Mean diffusivity
DP: Delayed phase
DWI: Diffusion-weighted imaging
ECM: Extracellular matrix
ECV: Extracellular volume
EMVI: Extramural venous invasion
FOV: Field of view
HR-MRI: High-resolution magnetic resonance imaging
IC: Iodine concentration
ICC: Intraclass correlation coefficient
Kmean: Mean kurtosis
LI: Labeling index
MRI: Magnetic resonance imaging
MSI: Microsatellite
RC: Rectal cancer
ROC: Receiver operating characteristic
ROI: Region of interest
TE: Echo time
TR: Repetition time
VMI: Virtual mono-energetic image
VP: Venous phase
