# Supplementary material for: Performance of dual-layer spectral CT-based extracellular volume fraction to assess Ki-67 proliferation status in rectal cancer: comparison with diffusion kurtosis imaging
Source: Insights Imaging. 2026 Aug 27;17:211. doi: 10.1186/s13244-026-02376-4 (PMC13522283; doi:10.1186/s13244-026-02376-4)
Supplement: Supplementary file 1 [file 13244_2026_2376_MOESM1_ESM.pdf]

Performance of dual-layer spectral CT-based extracellular volume fraction to assess Ki-67 proliferation status in rectal cancer: comparison with diffusion kurtosis imaging

ELECTRONIC SUPPLEMENTARY MATERIAL

Supplementary Table 1: Inter-observer and intra-observer reliability of IC_ tumor, IC_ artery, ECV, K_{mean}, D_{mean}, and ADC_{mean} values

	Inter-observer reliability		Intra-observer reliability	
	ICC (95% CI)	*p* value	ICC (95% CI)	*p* value
IC_tumor	0.880 (0.825–0.920)	< 0.001	0.831 (0.750–0.890)	< 0.001
IC_artery	0.986 (0.966–0.995)	< 0.001	0.973 (0.934–0.989)	< 0.001
ECV	0.905 (0.860–0.938)	< 0.001	0.861 (0.795–0.910)	< 0.001
K_{mean}	0.932 (0.875–0.964)	< 0.001	0.922 (0.887–0.945)	< 0.001
D_{mean}	0.853 (0.732–0.922)	< 0.001	0.860 (0.806–0.913)	< 0.001
ADC_{mean}	0.844 (0.713–0.912)	< 0.001	0.831 (0.720–0.905)	< 0.001

Abbreviations: ECV, Extracellular volume; ICC, Intraclass correlation coefficient; 95% CI, 95% Confidence interval.

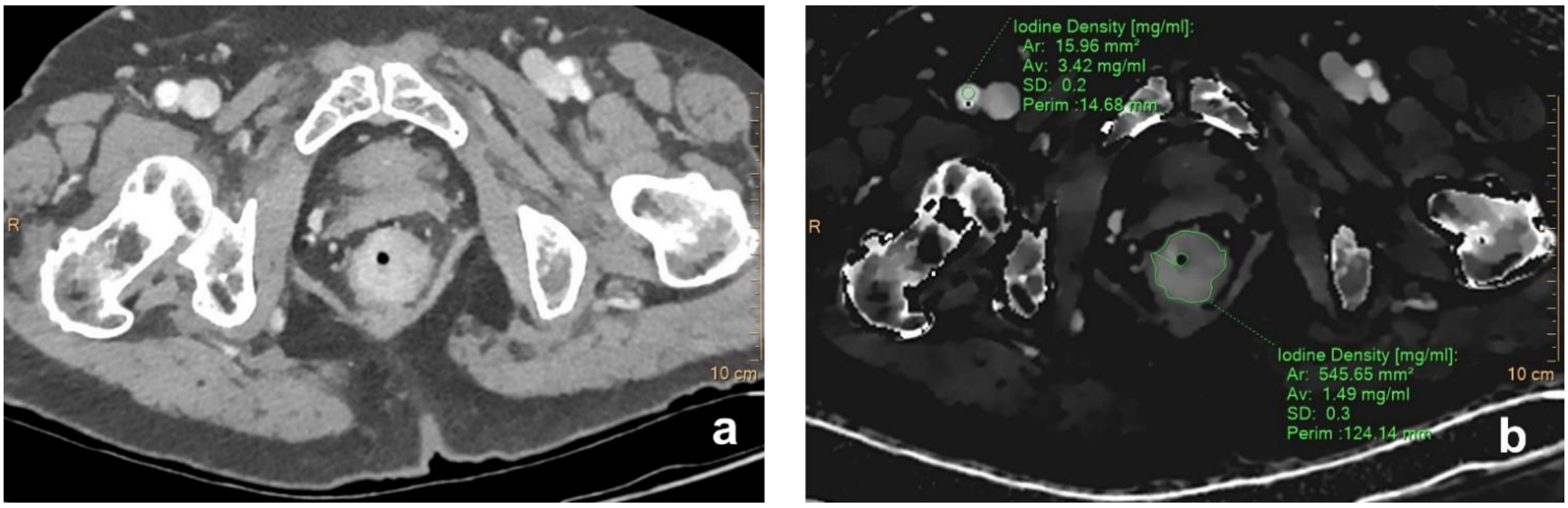

Supplementary Fig. 1 (a) 40-keV virtual monoenergetic images (VMI) from delayed phase (DP) dual-layer spectral CT (DLSCT) in a case of rectal cancer; (b) DP-iodine map, showing the delineated region of interest (ROI) of the rectal cancer tumor and a circular ROI placed in the ipsilateral external iliac artery. The measured iodine concentration (IC) values were 1.49 for the tumor and 3.42 for the artery, which were used for extracellular volume (ECV) fraction calculation.
